# Fertility Preservation in Pediatric Oncology: A 10-Year Single-Center Experience in Northern Spain

**DOI:** 10.3390/jcm14165762

**Published:** 2025-08-14

**Authors:** Anabel Carmona-Nunez, Maria Begoña Prieto Molano, Alba Gonzalez Lopez, Itziar Astigarraga, Ricardo Lopez-Almaraz

**Affiliations:** 1 Pediatric Oncology Group, Biobizkaia Health Research Institute, 48903 Barakaldo, Bizkaia, Spain; 2 Assisted Reproduction Unit, Department of Obstetrics and Gynecology, Cruces University Hospital, 48903 Barakaldo, Bizkaia, Spain; 3 Department of Radiotherapeutic Oncology, Cruces University Hospital, 48903 Barakaldo, Bizkaia, Spain; 4 Pediatric Hematology and Oncology, Department of Pediatrics, Cruces University Hospital, 48903 Barakaldo, Bizkaia, Spain; 5 Department of Pediatrics, Faculty of Medicine, University of the Basque Country, UPV/EHU, 48940 Leioa, Bizkaia, Spain

**Keywords:** childhood cancer survivors, cryopreservation, fertility preservation, azoospermia, premature ovarian insufficiency, testicular biopsy

## Abstract

**Background/Objectives**: The aim of this study is to describe fertility preservation (FP) techniques performed over the last 10 years at a tertiary hospital in northern Spain in patients under 18 diagnosed with cancer. **Methods**: A retrospective medical record review was conducted for patients aged 0 to 18 years diagnosed between January 2014 and December 2023 in the Pediatric Oncology Unit at a university hospital. We evaluated patient characteristics, the timing of FP procedures, and potential risk factors for ovarian insufficiency and early azoospermia. Additionally, we assessed the agreement between two gonadotoxicity risk classifications. **Results**: In our center, FP is more frequently offered to pubertal patients (12 to 16 years old), prior to treatment in those at high risk of subsequent gonadotoxicity (>80%), and after treatment in those at low risk (<20%). Additionally, the increased provision of FP over the last five years of the study suggests improved clinician uptake of this long-term effect of cancer treatment. Our study found weak agreement between available gonadotoxicity risk classifications, complicating the identification of FP candidates. Long-term follow-up of survivors allowed for the detection of ovarian insufficiency (1.2%) and early azoospermia (0.7%), enabling hormone replacement therapy when necessary. Hematopoietic stem cell transplantation (HSCT) emerged as a predictor of early infertility. **Conclusions**: Our study highlights the prevalence of gonadotoxicity in pediatric cancer patients at our center and the increasing access to FP techniques. The findings emphasize the importance of personalized medicine, tailored FP strategies based on individual risk, and long-term follow-up to assess fertility status.

## 1. Introduction

Over recent decades, advances in the treatment of childhood cancer have significantly improved survival rates. In Spain, the 5 year overall survival rate for pediatric cancer was 54.8% in the 1980s, rising to 84% by 2017, according to the most recent report from the National Registry of Childhood Tumors (RETI-SEHOP) [1]. The quality of life and long-term effects of antineoplastic therapy have become increasingly relevant, underscoring the importance of ongoing follow-up care for childhood cancer survivors.

Among these late effects, gonadotoxicity represents a major concern [2,3]. In males, infertility is primarily characterized by early azoospermia due to the damage to spermatogonia, the cells responsible for gamete production [4,5]. In females, it manifests as primary ovarian insufficiency (POI), resulting from both qualitative and quantitative depletion of the ovarian follicular reserve. The cumulative incidence of POI among long-term childhood cancer survivors has been reported to reach approximately 8% by the age of 40, which is significantly higher than that observed in their unaffected siblings [6].

Therefore, assessing the risk of future infertility at the time of cancer diagnosis is crucial to determine whether fertility preservation (FP) strategies should be considered [3].

Risk stratification tools have been developed to evaluate the likelihood of treatment-induced gonadotoxicity based on tumor type and therapeutic regimen [4,7,8]. The most gonadotoxic interventions include bilateral gonadal surgery, myeloablative conditioning prior to hematopoietic stem cell transplantation (HSCT), pelvic or gonadal radiotherapy (RT), and the administration of high-dose alkylating agents [3,4,8].

Fertility preservation should also be considered for patients with relapsed or secondary malignancies who require intensified, and potentially more gonadotoxic, treatments [9]. Furthermore, the risk of central hypogonadism must be evaluated in patients with brain tumors undergoing cranial or craniospinal RT or surgery involving the pituitary gland. In particular, a maximum dose (Dmax) ≥ 40 Gy to the hypothalamus or pituitary gland has been associated with impaired gonadotropin production and consequent gonadal dysfunction [3,10].

Current guidelines recommend offering FP to patients with an estimated infertility risk greater than 50% and a realistic probability of surviving at least five years [8,11]. The selection of FP techniques depends primarily on the patient’s age and pubertal status. In prepubertal girls, ovarian tissue cryopreservation (OTC) is considered an acceptable and established option [12], whereas testicular tissue cryopreservation (TTC) remains an emerging option for prepubertal boys [3]. OTC is considered a safe and effective procedure, associated with low complication rates and minimal delays in cancer treatment [9]. Although data on children under five years of age are limited [4], successful live births have been reported following prepubertal OTC, with more than 130 live births documented to date [13]. In contrast, TTC remains experimental, and no live births have been documented to date [9,14]. Due to its surgical nature and the limited amount of tissue that can be harvested, TTC is typically reserved for prepubertal boys at high risk of gonadotoxicity [15,16].

In adolescents and young adults, established FP options include sperm cryopreservation and oocyte cryopreservation. In females, oocyte cryopreservation (OC) becomes feasible after the onset of puberty and involves hormonal stimulation to retrieve mature oocytes, typically requiring a 2–3 weeks delay in treatment initiation. Therefore, OC is generally recommended for clinically stable patients or for those at high risk of relapse who have completed first-line therapy with a low or moderate risk of gonadotoxicity [3,11]. Conversely, sperm cryopreservation is a non-invasive and widely accessible technique that should be offered to all pubertal or postpubertal males who are concerned about future fertility [3].

The objective of this study is to describe the fertility preservation strategies implemented over the past 10 years in a Spanish tertiary hospital, which treats a median of 55 new pediatric cancer cases annually in patients aged 0–17 years. Specifically, we aimed to evaluate the characteristics of patients offered FP, the timing of these interventions during oncologic treatment, and potential risk factors for ovarian failure and early azoospermia in our cohort. Additionally, we analyzed the level of agreement between two widely used gonadotoxicity risk classification systems in routine clinical practice [7,8].

## 2. Materials and Methods

### 2.1. Participants and Ethics

A retrospective observational study was conducted at a tertiary hospital in northern Spain (Bilbao). The study protocol was approved by the Research Ethics Committee of the affiliated university hospital (protocol code E24/08).

Inclusion criteria: patients aged 0–17 years who were diagnosed with cancer between January 2014 and December 2023 at the Pediatric Hematology and Oncology Unit of the hospital.

Exclusion criteria: patients diagnosed with non-malignant hematologic conditions or benign tumors; those with a very low 5-year survival probability at diagnosis; patients with a known genetic syndrome prior to cancer diagnosis; patients referred from other institutions for follow-up care after receiving treatment elsewhere; and patients who were diagnosed at our center but subsequently referred to another hospital for treatment and follow-up.

### 2.2. Data Collection

A retrospective review of electronic medical records was conducted for all eligible patients. Demographic, clinical, and fertility-related data—including information on fertility preservation (FP) interventions—were systematically extracted.

Patients were stratified into three age groups according to pubertal status: prepubertal (<12 years), peripubertal (12–16 years), and postpubertal (>16 years) [16].

The risk of gonadal damage was assessed using two validated classification systems: the 2005 classification by Wallace et al. [7] and the 2021 Spanish consensus classification by Santaballa et al. [8]. For malignancies not explicitly addressed in these systems, exposure to alkylating agents was quantified using the validated Cyclophosphamide Equivalent Dose (CED) metric [17] and categorized according to the 2024 risk classification proposed by Talbot et al. [4].

Patients who had undergone pituitary surgery, had a diagnosis of hypopituitarism, or had received cranial or craniospinal radiotherapy with a maximum dose (Dmax) ≥ 40 Gy to the hypothalamus or pituitary gland were classified as high risk for hypogonadotropic hypogonadism [9,18].

Radiotherapy-related gonadal risk was defined as follows: a Dmax ≥ 4 Gy to the testes in males; a Dmax ≥ 15 Gy to the ovaries in prepubertal females; and a Dmax ≥ 10 Gy to the ovaries in postpubertal females [4,9].

Semen analysis results were categorized as follows: normospermic if sperm count was >15 million/mL with >40% motility; oligo-/asthenospermic if spermatozoa were present but outside these parameters; and azoospermic if no spermatozoa were detected [5].

Early primary ovarian insufficiency (POI) in females was diagnosed based on the presence of amenorrhea for at least four months and follicle-stimulating hormone (FSH) levels > 30 IU/L [19,20,21]. In these patients, the serum level of anti-Müllerian hormone (AMH), when available, was considered to assess its potential relationship with ovarian reserve [22,23,24]. In cases where FSH data were unavailable, POI was diagnosed based on an anti-Müllerian hormone (AMH) level < 0.1 ng/mL and ultrasonographic evidence of ovarian atrophy [22,23,24,25,26]. Although AMH and ultrasound are not currently included in the formal diagnostic criteria for POI, they are reliable indicators of ovarian reserve.

In males, early infertility was defined as either azoospermia or severe oligospermia on semen analysis [4,5].

### 2.3. Statistical Analysis

All data were anonymized and compiled using IBM SPSS Statistics (version 21, Chicago, EEUU). Statistical analyses were conducted using R software (version 4.3.3; R Core Team, 2024, New Zealand).

For qualitative variables, absolute and relative frequencies were reported. For quantitative variables, the mean and standard deviation (SD) were calculated when data followed a normal distribution; otherwise, the median and interquartile range (IQR) were reported. The Shapiro–Wilk test was used to assess the normality of quantitative variables.

Group comparisons for qualitative variables were performed using the Chi-square test or Fisher’s exact test, as appropriate. For quantitative variables, Student’s *t*-test was applied when distributions were normal, while the Kruskal–Wallis test was used for non-normally distributed variables.

Agreement between the two gonadotoxicity risk classification systems was assessed using Cohen’s kappa coefficient with quadratic weighting [27]. To identify potential risk factors associated with azoospermia or early POI, univariate logistic regression analyses were conducted, and model discrimination was evaluated using the area under the receiver operating characteristic (ROC) curve [28].

## 3. Results

### 3.1. Characteristics of Patients

**Figure 1:**
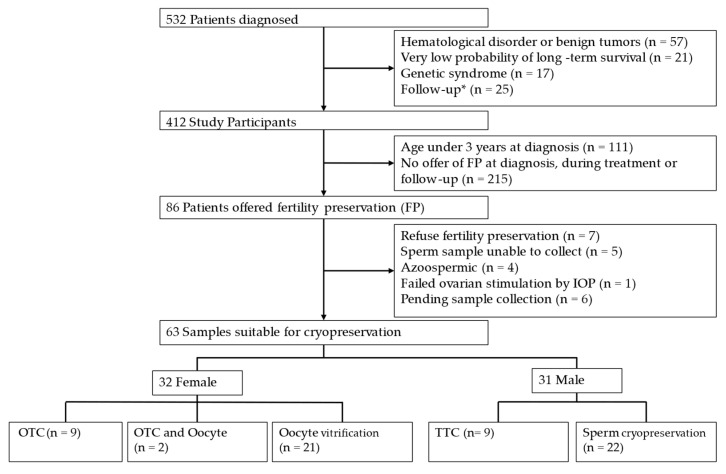
Flow diagram. * Patients treated elsewhere who start follow-up in our center or diagnosed here but referred elsewhere for treatment and follow-up. OTC: Ovarian tissue cryopreservation. TTC: Testicular tissue cryopreservation.

Between January 2014 and December 2023, a total of 532 patients aged 0 to 17 years were diagnosed in the Pediatric Hematology and Oncology Unit of our university hospital (Figure 1).

The lower rate of fertility preservation offers could be due to factors such as limited availability of techniques early in the study period, patients’ clinical condition at diagnosis, poor prognosis in some cases, and tumor types associated with a low risk of treatment-related infertility.

**Figure 2:**
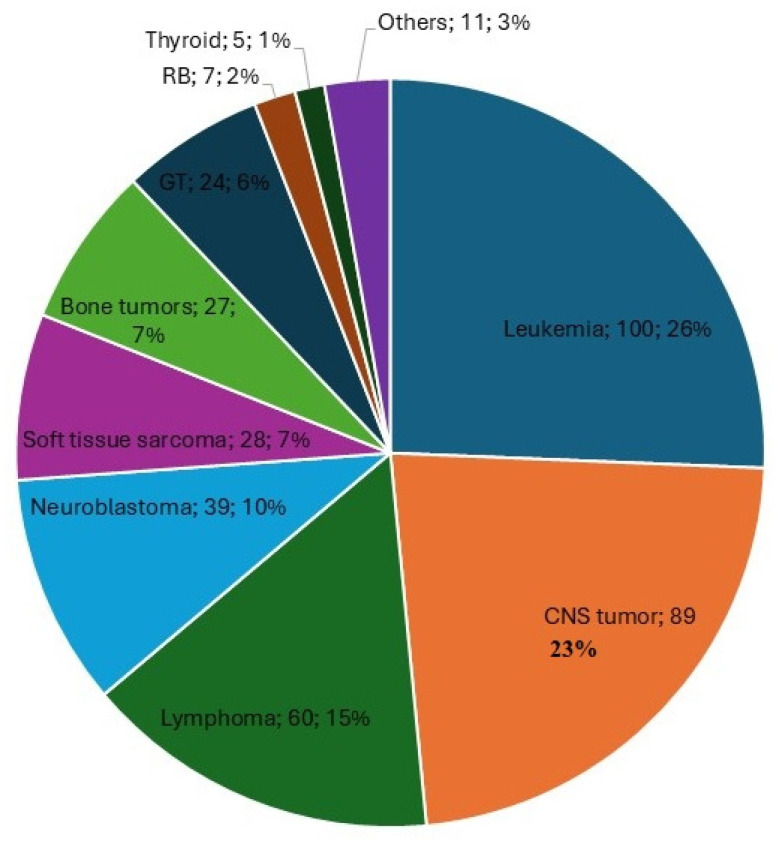
Primary diseases of children and adolescents included in the study. This figure includes the tumor type, the total number of patients diagnosed with that tumor, and the corresponding percentage relative to the total number of patients. CNS: Central nervous system and intracranial tumors, GT: Gonadal tumors. RB: retinoblastoma.

The median age at diagnosis was 6 years (interquartile range [IQR]: 2–12 years). A male predominance was observed, with 230 of 412 patients being male (56%). The most frequent tumor group was extracranial solid tumors (*n* = 163/412, 39.6%), followed by hematologic malignancies (*n* = 160/412, 38.8%) and central nervous system (CNS) and intracranial tumors (*n* = 89/412, 21.6%). The baseline characteristics of the patients at diagnosis are summarized in Table 1 and depicted in Figure 2.

### 3.2. Patient Characteristics at Study

Table 2 shows the characteristics of the patients as of December 2023, at the conclusion of the patient inclusion period in the study.

### 3.3. Risk of Infertility Associated with Antineoplastic Treatment

**Figure 3:**
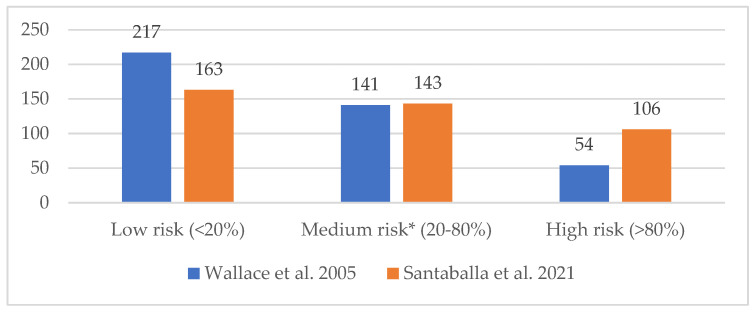
Risk of infertility associated with antineoplastic treatment (*n* = 412), according to the classification by Wallace et al. [7] and Santaballa et al. [8]. * In Santaballa et al. consensus [8], the medium-risk category is subdivided into two subcategories: low–intermediate risk (20–50% decreased probability of pregnancy or increased risk of infertility), with 123 patients in our population; intermediate–high risk (50–80% decreased probability of pregnancy or increased risk of infertility), with 20 patients in our population.

Figure 3 illustrates the risk of infertility in patients according to the 2005 classification by Wallace et al. [7] and the 2021 Spanish consensus by Santaballa et al. [8].

We assessed the degree of agreement between both risk classifications [7,8] using a Cohen’s kappa coefficient with quadratic error weighting, which revealed a weak agreement (0.40 ≤ κCohen ≤ 0.59) [27]. Consequently, we opted to establish the gonadotoxicity risk for our patients based on the 2021 Spanish consensus classification [8], as it aligns with the treatment protocols at our institution and is more current than the 2005 classification by Wallace et al. [7].

Only a small proportion of patients were categorized at high risk for hypogonadotropic hypogonadism (*n* = 18/412, 4.4%). This was mainly due to hypopituitarism (*n* = 15/18, 83%), pituitary gland surgery (*n* = 13/18, 66.6%), or cranial/pineal RT with a Dmax ≥ 40 Gy to the pituitary gland (*n* = 2/18, 11%).

Gonadal RT dose was identified as a high-risk factor for gonadotoxicity in most of the patients (*n* = 13/16, 81%). Gonadal surgery was performed in 26 patients (*n* = 26/412, 6.3%), though only one required bilateral gonadal removal (*n* = 1/412, 0.24%). Additionally, 9.5% of patients (*n* = 39/412) required HSCT, and three patients (*n* = 3/412, 0.7%) underwent chimeric antigen receptor T-cell (CART) therapy.

### 3.4. Fertility Preservation

Fertility preservation options were offered to 20.8% of patients diagnosed with tumors in childhood at our center (*n* = 86). The majority of these patients were able to provide a sample suitable for cryopreservation (*n* = 63/86, 73.3%). Fertility preservation was offered equally to males (*n* = 44/86, 51.2%) and females (*n* = 42/86, 48.8%).

Among those who visited the reproductive medicine specialist (*n* = 79), serology for CMV, HIV, and hepatitis B/C was negative prior to sample collection in most patients (*n* = 66/79, 83.5%). In cases where a positive result was obtained, samples were stored separately to prevent contamination. Hormonal studies were performed at diagnosis in 30 patients (*n* = 30/79, 38%), with AMH levels measured in half of the female adolescents (*n* = 10/20, 50%). The median AMH level at diagnosis was 1.94 ng/mL (SD = 1.06).

We analyzed the association between the offer of fertility preservation and various clinical variables (Table 3). A statistically significant relationship was found between the gonadotoxicity risk group and the offer of fertility preservation (*p* < 0.001), with a higher frequency of offers in high-risk patients (*n* = 33/86, 38.4%). No significant differences were observed in the offer of fertility preservation based on tumor group (*p* = 0.328) or with regard to gonadal RT as part of treatment (*p* = 0.08).

#### 3.4.1. Patient Age

A statistically significant association was found between the offer of fertility preservation and the age group at diagnosis (*p* < 0.001) (see Table 3), with offers being more frequent in patients older than 12 years old (*n* = 60/86, 70%).

A significant association was also found between the age group at fertility preservation and the technique used (*p* < 0.001) (see Table 4). In the prepubertal age group (<12 years), the predominant technique was OTC (57.1%, *n* = 8/14). In the peripubertal (12–16 years) group, sperm cryopreservation was the most common technique in men (35.6%, *n* = 16/45), whereas in the postpubertal (>16 years) group, oocyte cryopreservation following ovarian hormonal stimulation predominated in women (35%, *n* = 7/20).

#### 3.4.2. Time of Fertility Preservation

FP strategies at our center evolved over time: In 2017, a formal protocol for semen cryopreservation in pubertal males was implemented; in 2018, testicular tissue cryopreservation was introduced as part of a research initiative; oocyte cryopreservation became available on-site in 2015 through multidisciplinary coordination; and ovarian tissue cryopreservation, authorized since 2010, was first performed in a prepubertal girl in 2017. Prior to these dates, FP techniques could be offered to selected patients, but procedures were carried out at a referral hospital located outside our autonomous community

FP was most frequently offered prior to the initiation of antineoplastic treatment (59.3%, *n* = 51/86). The remaining patients were offered FP after the completion of antineoplastic treatment (23.3%, *n* = 20/86), during treatment (9.3%, *n* = 8/86), both before and after treatment (2.3%, *n* = 2/86), or at the time of diagnosis of relapsed disease or second malignancies (5.8%, *n* = 5/86). Some patients underwent FP more than four years after their cancer diagnosis (16.4%, *n* = 13/86); all of them had been diagnosed during the first five years of the study period (2014–2018).

A statistically significant association was found between the timing of FP and the risk of gonadotoxicity [8] (p 0.007), as detailed in Table 5. Among those who underwent FP prior to the initiation of antineoplastic treatment, the high-risk gonadotoxicity group predominated (41.2%, *n*= 21/51). In contrast, among those who underwent FP after treatment, most patients had a low risk of gonadotoxicity (55%, *n*= 11/20). All patients who underwent FP at the time of diagnosis of relapsed disease or second malignancies had a gonadotoxicity risk greater than 50% (100%, *n*= 5/5).

To evaluate trends in the offer of FP, patients were categorized into two subgroups based on the diagnostic period: the first five years of the study (2014–2018) and the last five years (2019–2023). A significant association was observed between the diagnosis period and the offer of FP (*p* 0.02), as well as between the diagnosis period and the timing of FP (*p* < 0.001). No significant differences were found between the type of FP technique performed across the two periods (*p* 0.11), as shown in Table 6.

During the second period, physicians offered FP more frequently (55.8%, *n* = 48/86), and samples suitable for cryopreservation were obtained more often (68.3%, *n* = 43/63). Regarding the timing of FP, among patients diagnosed in the first period, FP predominantly occurred after the completion of antineoplastic treatment (42.1%, *n* = 16/38), whereas in the second period, FP was more commonly offered prior to the initiation of treatment (75%, *n* = 36/48).

#### 3.4.3. Quality of the Samples Obtained and Complications

A total of 75 patients (87%, *n* = 75/86) underwent fertility preservation (FP) techniques. The majority experienced no complications related to the procedure (92%, *n* = 69/75). Only minor complications associated with oocyte cryopreservation (8%, *n* = 6/75) were recorded, which did not require additional measures, except analgesia. Among these complications, one patient experienced pain after the procedure, another had pain following multiple cycles of ovarian stimulation and repeated punctures, while four patients underwent several ovarian punctures without reporting pain associated with the technique. Table 7 outlines the quality of the samples obtained for fertility preservation.

### 3.5. Follow-Up After Treatment

Follow-up of childhood cancer survivors in the pediatric oncology department lasted between 4 and 10 years in most cases (58%, *n* = 239/412). During the follow-up period, 38 patients who had reached puberty were referred to a reproductive medicine specialist for fertility evaluation (9.2%, *n* = 38/412). Referrals were most common between 4 and 10 years after tumor diagnosis (52.6%, *n* = 20/38). The majority of those referred were adolescent or young adult females (65.8%, *n* = 25/38). The primary reason for referral was the consideration of fertility preservation (65.8%, *n* = 25/38), while the remainder were referred to assess their fertility status following oncological treatment (34.2%, *n* = 13/38). No patient requested the use of the preserved samples to have offspring during the study period.

A significant association was found between gonadotoxicity risk [8] and the assessment of reproductive status after cancer treatment (*p* < 0.05), as presented in Table 8.

In the gynecological assessment following antineoplastic treatment, AMH were evaluated in the majority of women (80%, *n* = 20/25), with a median of 1.1 ng/mL (IQR; 0.18–2.3). AMH levels were significantly lower in adolescents with primary ovarian insufficiency (POI) (*p* = 0.02).

**Figure 4:**
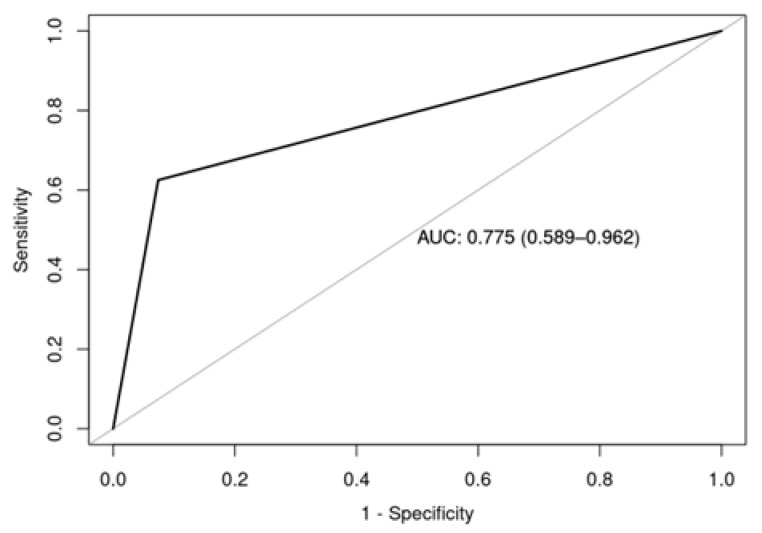
Area under the curve ROC of the univariate logistic regression model to identify factors present in patients with POI or early azoospermia.

Early infertility was diagnosed in eight patients (1.9%, *n* = 8/412). The mean age at diagnosis was 16.38 years (SD = 2.92). The majority of these patients were women with POI (63%, *n* = 5/8). Of the five women diagnosed with POI, four were receiving sex hormone replacement therapy (80%). In univariate logistic regression analysis to identify factors associated with POI or early azoospermia, hematopoietic stem cell transplantation (HSCT) was statistically significant (OR 20.83, 95% CI 3.13–205.8, *p* = 0.003). These results suggest that a patient who underwent HSCT is 20.83 times more likely to experience ovarian failure or early azoospermia compared to a patient who did not undergo HSCT. The area under curve ROC (AUC_ROC_) was 0.77, indicating that the diagnostic accuracy of the logistic regression model is acceptable (0.7 < AUC_ROC_ < 0.8) [28], as shown in Figure 4.

## 4. Discussion

Gonadotoxicity, a long-term effect of oncological treatments, has gained increasing attention in pediatric oncology due to its potential risks to fertility [4,8].

This study provides an overview of fertility preservation (FP) practices over the last decade in pediatric oncology patients at a tertiary hospital in northern Spain. The primary limitations of this study are its single-center design and the relatively smaller sample size compared to larger reference centers. Additionally, at the start of the study period, experimental techniques for prepubertal patients were introduced in our center, which limited experience with these methods in the early years of the study. Early in the study period, variability in clinical practice among physicians may have influenced FP decision-making, highlighting the importance of establishing standardized protocols and interdepartmental coordination. A further limitation is that our study did not evaluate institutional or systemic factors—such as guideline implementation or team training—that may have influenced the observed increase in FP uptake. Although follow-up visits were temporarily reduced during the COVID-19 pandemic, fertility preservation procedures remained available, and no significant disruptions in access or timing were observed for eligible patients. Despite these limitations, the results reflect a growing uptake among the healthcare team about the long-term effects of oncological treatments, as well as significant improvements in the provision of fertility preservation throughout the study period.

### 4.1. Characteristics of the Cohort

The findings of our study align with previous reports on the age and sex distribution of childhood cancer patients [1,29]. Although hematological tumors are the most common in national cancer registries [1,29], our cohort showed a predominance of extracranial solid tumors, followed by hematological malignancies. The presence of metastatic disease (13.6%) and relapses or second malignancies (18.4%) highlights the complexity of this patient population, which in turn influences fertility preservation decisions.

### 4.2. Risk of Gonadotoxicity and Fertility Preservation Techniques

The agreement between the gonadotoxicity risk classification proposed by Wallace et al. (2005) [7] and the Spanish consensus by Santaballa et al. (2021) [8] was weak. The classification by Talbot et al. (2024) [4], stratifies gonadotoxicity risk based on the treatment received, but it lacks a table correlating the most common tumor types with their associated gonadotoxicity risks based on typical therapeutic protocols. This omission limits the practical applicability of such classifications in clinical settings, as it necessitates a more time-consuming evaluation of the cumulative doses each patient will receive. Moreover, there is an ongoing challenge in that cancer treatments are continuously evolving, while FP techniques are advancing at a slower pace. As new therapeutic agents and protocols are developed, it becomes essential to assess their gonadotoxicity through longitudinal data to better understand their reproductive risks. Consequently, risk classifications may always carry inherent limitations, as gonadotoxicity data tend to lag behind information on long-term fertility outcomes. This underscores the need for future studies to develop a consensus and standardized classification system for identifying patients who are suitable candidates for fertility preservation.

In accordance with the existing literature [3,8], our study observed that fertility preservation was more frequently offered to pubertal patients, prior to treatment in those at high risk of gonadotoxicity (>80%) and at follow-up for patients at lower risk of infertility (<20%). In adolescents, the most commonly employed techniques were oocyte and sperm cryopreservation, whereas in prepubertal children, ovarian and testicular tissue cryopreservation were the only methods. These techniques were introduced in our center in 2017 and 2018, respectively, as part of a research initiative conducted by our hospital’s affiliated health research institute.

Regarding quality criteria for fertility preservation, preoperative infectious serology and hormonal assessments prior to FP procedures are recommended [4]. Additionally, the majority of patients in our study (60%) adhered to the recommendation of cryopreserving at least 10 to 15 oocytes [8].

Notably, fertility preservation was most commonly offered before the initiation of antineoplastic treatment (59%). This finding highlights the importance of early detection of gonadotoxicity risk at the time of diagnosis. Furthermore, a higher proportion of patients diagnosed in the latter half of the study period were offered fertility preservation, indicating increased uptake among the medical team at our center about the long-term reproductive risks of oncological treatments.

### 4.3. Long-Term Follow-Up and Outcomes

Although other studies have reported live births following fertility preservation techniques in the pediatric population [9,14,30], not enough time has elapsed to report the rate of utilization of preserved samples or live births in our cohort.

Referral to a reproductive medicine specialist during follow-up allowed us to identify cases of ovarian failure and early azoospermia, particularly in pubertal and young adult women. These patients exhibited significantly lower levels of AMH, which is consistent with early ovarian failure and reinforces its role as a valuable marker of ovarian reserve [22,23,24,25,26].

POI is often linked to early infertility, but it also results in hormonal deficits, such as estrogen deficiency, leading to long-term sequelae like osteoporosis and cardiovascular disorders [31]. Estrogen replacement therapy until the average age of menopause has been shown to reduce cardiovascular disease mortality in women with POI in the general population [21,32]. In our cohort, most women with POI (80%) received sex hormone replacement therapy in line with current recommendations.

### 4.4. Predictors of POI and Azoospermia

The conditioning treatment associated with hematopoietic stem cell transplantation (HSCT) was found to be a significant predictor of POI and early azoospermia, with an odds ratio of 20.83 according to the logistic regression model. This finding highlights the critical need for tailored fertility preservation strategies for patients in this high-risk subgroup.

## 5. Conclusions

The findings of this study underscore the importance of a multidisciplinary approach that integrates pediatric oncology and reproductive medicine to optimize long-term quality of life outcomes for childhood cancer survivors. Continued research into safe and effective fertility preservation techniques for prepubertal children and the development of predictive fertility markers remain essential.

In conclusion, our study highlights the need for individualized FP strategies based on each patient’s risk profile and emphasizes the importance of long-term fertility follow-up. Additionally, emerging national and international FP registries—such as UKSTORE in the UK—represent valuable tools for tracking FP uptake and outcomes, and can help improve risk classification through large-scale, collaborative datasets beyond the limitations of single-center studies.

This study provides a detailed overview of FP techniques applied over the past decade in a tertiary pediatric oncology center, demonstrating how FP practices have evolved and become more tailored according to individual patient risk factors. Our findings support the implementation of personalized FP strategies and highlight the critical role of systematic, long-term follow-up to monitor reproductive outcomes and inform future clinical decision-making.

## Figures and Tables

**Table 1:** Descriptive characteristics of patients at diagnosis (*n* = 412).

Age at Diagnosis	*n*	%
0–4 years	167	40.5
5–11 years	129	31.3
12–16 years	107	26
>16 years	9	2,2
Sex	*n*	%
Female	180	43.7
Male	230	55.8
Transgender	2	0.5
Metastasis	*n*	%
No	356	86.4
Yes	56	13.6

**Table 2:** Descriptive characteristics of patients at study (*n* = 412).

Age at Study	*n*	%
0–4 years	31	7.5
5–11 years	139	33.7
12–16 years	100	24.3
>16 years	101	24.5
Deceased	41	10
Pubertal status	*n*	%
Prepubertal	177	43
Normal puberty	135	32.8
Late puberty	10	2.4
Early puberty	7	1.7
Sex hormone replacement therapy	9	2.2
Transgender-induced puberty	1	0.2
No pubertal records *	32	7.7
Deceased	41	10
Relapsed disease or 2nd malignancies	*n*	%
No	336	81.6
Yes	76	18.4
Current status	*n*	%
Alive, no evidence of disease	324	78.7
Alive with intractable tumor **	10	2.4
Alive, on-treatment	37	8.9
Deceased	41	10

* No hormonal or clinical puberty data have been recorded in the electronic medical record ** Includes patients with brain tumors who did not receive treatment, only monitoring through imaging tests, or those who, after receiving treatment, have residual tumor tissue and are followed up with imaging to assess progression, without the need for further treatment.

**Table 3:** Bivariate table relating fertility preservation offer to variables of interest.

	All (*n* = 412)	No (*n* = 326)	Yes (*n* = 86)	*p*
Gonadotoxicity risk [8], *n* (%)				<0.001
Low	163 (39.6%)	143 (43.9%)	20 (23.3%)	
Low–intermediate	123 (29.85%)	100 (30.7%)	23 (26.7%)	
Intermediate–high	20 (4.85%)	10 (3.1%)	10 (11.6%)	
High	106 (25.7%)	73 (22.3%)	33 (38.4%)	
Age group at diagnosis, *n* (%)				<0.001
Prepubertal (<12 y)	296 (71.8%)	270 (82.8%)	26 (30.2%)	
Peripubertal (12–16 y)	107 (26%)	55 (16.9%)	52 (60.5%)	
Postpubertal (>16 y)	9 (2.2%)	1 (0.3%)	8 (9.3%)	
Tumor group, *n* (%)				0.328
Extracranial solid	163 (39.6%)	129 (39.6%)	34 (39.53%)	
Hematologic	160 (38.83%)	122 (37.42%)	38 (44.19%)	
CNS	89 (21.6%)	75 (23.01%)	14 (16.28%)	
Gonadal or pelvic RT, *n* (%)				0.085
No	398 (96.6%)	318 (97.5%)	80 (93%)	
Yes	14 (3.4%)	8 (2.5%)	6 (7%)	

CNS: Central nervous system and intracranial tumors. RT: radiation therapy. Y: years.

**Table 4:** Bivariate table relating age group at fertility preservation to fertility preservation options.

	All	<12 y	12–16 y	>16 y	*p*
FP Options, *n* (%)	*n* = 79	*n* = 14	*n* = 45	*n* = 20	<0.001
OTC	9 (11.4%)	7 (50%)	2 (4.4%)	0 (0%)	
OTC and Oocyte *	2 (2.5%)	1 (7.1%)	1 (2.2%)	0 (0%)	
Oocyte cryopreservation	21 (26.6%)	0 (0%)	14 (31.1%)	7 (35%)	
TTC	9 (11.4%)	6 (42.9%)	3 (6.7%)	0 (0%)	
Sperm cryopreservation	22 (27.9%)	0 (0%)	16 (35.6%)	6 (30%)	
Sample unable to collect	10 (12.7%)	0 (0%)	6 (13.3%)	4 (20%)	
Pending sample to collect	6 (7.6%)	0 (0%)	3 (6.7%)	3 (15%)	

* At diagnosis, a sample of ovarian tissue was cryopreserved. After treatment, the patient underwent ovarian stimulation with oocyte cryopreservation. FP: fertility preservation; OTC: ovarian tissue cryopreservation; TTC: testicular tissue cryopreservation; y: years.

**Table 5:** Bivariate table relating the timing of preservation to the risk of gonadotoxicity.

	All (*n* = 86)	Pre- Treatment (*n* = 51)	During Treatment (*n* = 8)	Post- Treatment (*n* = 20)	Pre and Post Treatment (*n* = 2)	Relapse * (*n* = 5)	*p*
**Gonadotoxicity** **Risk** [8], ***n* (%)**							0.007
Low	20 (23.3%)	8 (15.7%)	1 (12.5%)	11 (55%)	0 (0%)	0 (0%)	
Low–intermediate	23 (26.7%)	14 (27.4%)	2 (25%)	6 (30%)	1 (50%)	0 (0%)	
Intermediate–high	10 (11.6%)	8 (15.7%)	1 (12.5%)	1 (5%)	0 (0%)	0 (0%)	
High	33 (38.4%)	21 (41.2%)	4 (50%)	2 (10%)	1 (50%)	5 (100%)	

* Relapse or second malignancies.

**Table 6:** Evolution of fertility preservation offer according to diagnosis period.

	All	2014–2018 (*n* = 230)	2019–2023 (*n* = 182)	*p*	*n*
FP offer, *n* (%)				0.02	412
No	326 (79.1%)	192 (83.5%)	134 (73.6%)		
Yes	86 (20.9%)	38 (16.5%)	48 (26.4%)		
Time of FP, *n* (%)				<0.001	86
Pretreatment	51 (59.3%)	15 (39.5%)	36 (75%)		
During treatment	8 (9.3%)	3 (7.9%)	5 (10.4%)		
Post-treatment	20 (23.3%)	16 (42.1%)	4 (8.3%)		
Pre- and post-treatment	2 (2.3%)	0 (0%)	2 (4.2%)		
Relapse or second malignancies	5 (5.8%)	4 (10.5%)	1 (2.1%)		
FP options, *n* (%)				0.11	73
OTC	9 (12.3%)	2 (7.4%)	7 (15.2%)		
OTC and Oocyte *	2 (2.7%)	0 (0.00%)	2 (4.35%)		
Oocyte cryopreservation	21 (28.8%)	10 (37%)	11 (23.9%)		
TTC	9 (12.3%)	2 (7.4%)	7 (15.2%)		
Sperm cryopreservation	22 (30.1%)	6 (22.2%)	16 (34.8%)		
Sample unable to collect	10 (13.7%)	7 (25.9%)	3 (6.5%)		

* At diagnosis, a sample of ovarian tissue was cryopreserved. After treatment, the patient underwent ovarian stimulation with oocyte cryopreservation. FP: fertility preservation; OTC: ovarian tissue cryopreservation; TTC: testicular tissue cryopreservation.

**Table 7:** Quality of the samples obtained.

Cryopreserved Oocytes	*n*	%
<10 oocytes	9	39.2
10–15 oocytes	7	30.4
>15 oocytes	7	30.4
Semen analysis	*n*	%
Normospermia	15	65.2
Oligo/asthenospermia	4	17.4
Azoospermia	4	17.4

**Table 8:** Bivariate table relating post-treatment fertility assessment to gonadotoxic risk.

	All (*n* = 38)	Fertility Assessment * (*n* = 13)	FP ** (*n* = 25)	*p*
**Gonadotoxicity Risk** [8]**, *n* (%)**				**0.01**
Low	12 (31.6%)	1 (7.7%)	11 (44%)	
Low–intermediate	7 (18.4%)	1 (7.7%)	6 (24%)	
Intermediate–high	3 (7.9%)	2 (15.4%)	1 (4%)	
High	16 (42.1%)	9 (69.2%)	7 (28%)	

* Patients referred to a reproductive medicine specialist for fertility evaluation after completing oncological treatment. ** Patients referred to a reproductive medicine specialist for FP evaluation after completing oncological treatment.

## Data Availability

Data is unavailable due to privacy or ethical restrictions.
